# Transforming pandemic influenza antiviral stockpiling using a capacity-based approach: A modeling study

**DOI:** 10.1371/journal.pmed.1004992

**Published:** 2026-11-03

**Authors:** Nir Gavish, Amit Huppert

**Affiliations:** 1 Faculty of Mathematics, Israel Institute of Technology, Haifa, Israel; 2 Data & Analytics, Sheba Medical Center, Ramat Gan, Israel; 3 Department of Epidemiology and Preventive Medicine, the Gray Faculty of Medical & Health Sciences, Tel Aviv University, Tel Aviv-Yafo, Israel; University Medical Centre Utrecht: Universitair Medisch Centrum Utrecht, NETHERLANDS, KINGDOM OF THE

## Abstract

**Background:**

Pandemic influenza preparedness in high-resource nations has traditionally relied on large antiviral stockpiles, typically targeting coverage for 25% of the population. This inventory-based benchmark focuses on cumulative disease burden but often overlooks the inelastic capacity of healthcare systems to manage acute surges. As multi-billion-dollar inventories reach expiration, we evaluated whether static stockpiles remain a viable strategy for preventing healthcare system collapse during future pandemics.

**Methods and findings:**

We projected daily hospital bed demand using three complementary frameworks: a static lower-bound estimation, a dynamic deterministic Susceptible-Infectious-Hospitalized-Recovered transmission model, and historical reconstructions of the 1918, 1957, and 1968 pandemics using mortality data. We evaluated official planning scenarios for the United Kingdom, Japan, and the United States, comparing projected demand against national surge capacities (i.e., staffed beds). To test the theoretical limits of the stockpile strategy, we applied best-case assumptions, including perfect distribution of antivirals to 100% of symptomatic cases and high clinical effectiveness (30% to 60% reduction in hospitalization).

Our models identified a critical feasibility gap. While stockpiles successfully suppressed demand to manageable levels in moderate scenarios (analogous to 1957/1968), they were structurally insufficient for severe events. In high-severity scenarios (analogous to 1918 or CDC Very Severe parameters), the daily surge of patients exceeded available hospital capacity by a factor of 5–9. This inundation occurred even under the assumption of 100% treatment coverage, rendering antivirals incapable of preventing transition to crisis standards of care. Although this analysis is subject to the inherent uncertainties of future pandemics, our use of optimistic best-case assumptions suggests that the identified structural failures are a lower-bound estimate. Realistic operational hurdles would only widen the disparity between stockpile targets and system capacity.

The main limitations of this study’s methodology are its inherent optimistic bias, specifically the use of a conservative 4-day hospital length of stay that underestimates prolonged intensive care needs, the reliance on general hospital beds as a proxy that abstracts away from critical sub-system bottlenecks, and the assumption of guaranteed antiviral efficacy against novel strains.

**Conclusions:**

This study demonstrates that large reserves cannot overcome the rigid physical constraints of healthcare systems, even under the optimistic assumption of perfect distribution. Consequently, the conventional 25% coverage target for antiviral stockpiles, a hallmark of legacy planning, presents a critical opportunity for re-evaluation. As global stocks near expiration, policymakers are uniquely positioned to leverage this inflection point by exploring a capacity-based framework. This adaptive paradigm suggests incorporating a synergistic mix of targeted reserves, temporary non-pharmaceutical interventions, and flexible delivery options, such as decentralized home-first care. Together, these integrated measures could serve as a vital temporal bridge, preserving system integrity until vaccine deployment.

## Author summary

**Why was this study done?:**

- As massive global pandemic influenza stockpiles reach their expiration dates, governments worldwide face an imminent, multi-billion-dollar decision on whether to simply renew these existing reserves.
- Preparedness in many high-income nations relies heavily on inventory-based benchmarks, such as mandating antiviral stockpiles (e.g., oseltamivir) to cover 25% of the population.
- These traditional benchmarks are calculated based on the total expected number of infections (cumulative burden), but they crucially ignore the actual daily limits of hospital beds and available medical staff (capacity constraints).
- We conducted this research to determine whether relying on these static, inventory-based stockpiles is actually sufficient to prevent health system collapse during moderate, severe and very severe pandemics.

**What did the researchers do and find?:**

- We modeled daily hospital bed demand for the US, UK, and Japan using official planning scenarios and historical data from the 1918, 1957, and 1968 pandemics.
- To test the theoretical best-case limits of current strategies, we assumed antivirals were perfectly distributed to 100% of symptomatic patients immediately and had high effectiveness in preventing hospitalization.
- Even under these ideal conditions, we identified a critical feasibility gap during severe pandemics, where patient surges exceeded national hospital capacity by a factor of 5–9.
- Our analysis demonstrates that while stockpiles are potentially effective for moderate outbreaks, they are structurally incapable of preventing healthcare system collapse during high-severity events.

**What do these findings mean?:**

- The current strategy provides an illusion of systemic resilience for severe pandemics; having enough antivirals to treat 25% of the population, as a standalone policy, will not prevent health system collapses.
- It is important for policymakers to shift from inventory-based planning to incorporate capacity-based approaches in future planning of stockpiles.
- The main limitations of this study are that it assumes a best-case scenario with perfect drug distribution and effectiveness, and does not account for prolonged intensive care needs or specialized staff shortages.

## Abstract 3

In this modeling study, Nir Gavish and Amit Huppert examine whether traditional pandemic influenza antiviral stockpiles can keep hospital demand within available healthcare capacity during future pandemics. Using multiple modeling approaches and historical pandemic scenarios, they find that while stockpiles may help in moderate outbreaks, they are unlikely to prevent severe capacity pressures in high-impact scenarios, highlighting the potential value of preparedness strategies that place greater emphasis on healthcare system capacity and operational flexibility.

## Introduction

Pandemic influenza remains a persistent threat to global health security. The 20th century witnessed three major pandemics in 1918, 1957, and 1968, with the 1918 event alone claiming an estimated 50 million lives worldwide [1,2]. More recently, the 2009 H1N1 pandemic and the global devastation of COVID-19 have demonstrated that despite medical advancements, modern societies remain profoundly vulnerable to novel respiratory pathogens [3].

In response to this risk, particularly following the emergence of avian H5N1, high-resource nations invested billions in a specific form of insurance: the antiviral stockpile [4–8]. While contemporary preparedness frameworks feature an integrated suite of tools such as clinical prioritization and non-pharmaceutical interventions (NPIs), the strategic accumulation of neuraminidase inhibitors (e.g., oseltamivir) remains a central pillar. This strategy is predicated on the assumption that a novel virus will trigger a sudden wave of illness requiring immediate treatment to bridge the gap until vaccines become available [9,10]. As these massive inventories approach shelf-life expiration, governments face imminent decisions regarding multi-million dollar renewals [11–13].

Historically, stockpile targets have been guided by a 25% benchmark, the objective of securing sufficient courses to treat approximately one-quarter of the population [4–7]. While this figure became the international standard, some nations pursued even more aggressive targets; for example, the UK at one point amassed sufficient antivirals to cover 50% of its population [14]. These targets stem from epidemiological models estimating cumulative clinical attack rates over the course of a pandemic wave, together with broader considerations. The prevailing logic is straightforward and inventory-driven: if a specific number of individuals are projected to fall ill, the strategy mandates an equivalent number of treatment courses to mitigate the impact [4]. This paradigm is often refined by prioritization strategies, where stockpiling targets are narrowed to provide coverage only for specific high-risk groups rather than the general population.

However, this planning paradigm contains a critical omission, brought into sharp relief by the COVID-19 pandemic: it fails to account for the finite, inelastic capacity of the healthcare system. One of the most critical challenges of a severe pandemic is not merely the cumulative disease burden, but the acute, instantaneous surge of severe cases that triggers the functional collapse of hospital infrastructure, as observed in Lombardy, Italy and New York City in early 2020 [15–18]. By focusing exclusively on the *total* volume of treatments, the conventional model ignores the *rate* of demand, specifically, the daily requirement for staffed care beds.

Foundational modeling studies have extensively explored the dynamics of antiviral stockpiling in pandemic scenarios. Historically, this literature has focused on two primary areas: the optimal allocation of limited pharmaceutical inventories to minimize cumulative mortality [9], and the impact of maintaining specific population-coverage targets [10,19]. However, a critical gap remains. Traditional models frequently evaluate strategic success based on total cumulative disease burden, implicitly treating healthcare systems as elastic reservoirs capable of absorbing concentrated surges. Consequently, there is a lack of analytical frameworks that evaluate the viability of stockpiling strategies when constrained by the rigid, real-time limits of hospital capacity. To reconcile this disconnect, our study shifts the analytical focus from inventory optimization to capacity preservation.

This study evaluates the robustness of current antiviral strategies against the risk of healthcare system collapse. We employ parsimonious strategic models rather than complex predictive simulations to test the fundamental limits of the strategy under best-case conditions. By integrating standardized high-severity planning scenarios with historical reconstructions, we assess the central question of this study: whether massive static stockpiles remain a viable centerpiece for preventing the saturation of hospital capacity in the next global health crisis.

## Methods

### Study design and data sources

We employed strategic modeling to project daily hospital bed demand across varying pandemic severities. Specifically, we evaluated the effectiveness of large-scale antiviral distribution in mitigating surge capacity breaches and maintaining healthcare system continuity. See S1 Appendix for details.

We deliberately adopted a parsimonious modeling approach, as highly detailed frameworks often prove less reliable under the extreme uncertainty of future pandemic transmission dynamics. Hyper-parameterized models are notoriously susceptible to overfitting, which frequently leads to unreliable or misleading projections, a challenge documented in analyses of population-level resurgence and age-group clustering [20,21]. Fine-grained social contact networks serve as a clear example of this vulnerability: as the COVID-19 pandemic illustrated, these networks are rarely static, shifting fundamentally in response to behavioral changes and public health interventions [20–22]. Within our framework, incorporating such complexity is not only mathematically risky but also structurally unnecessary. Because we assume an unlimited stockpile distributed to 100% of symptomatic cases, the need to simulate treatment prioritization, and the detailed demographic or social structures required to support it, is effectively eliminated. Our use of optimistic best-case assumptions provide lower-bound estimates. As we will show, our results show systematic failure even under those idealized conditions. Relaxation of those assumptions, e.g., by accounting for realistic operational hurdles or limited stockpile size, would only widen the disparity between stockpile targets and system capacity.

To evaluate the universality of capacity constraints, we utilized healthcare system data and official pandemic influenza plans from the United Kingdom [6], the United States [7], and Japan [23] as high-resource case studies. While the qualitative patterns of system saturation were consistent across all three settings, the main text prioritizes the analysis of the United States to provide a focused narrative. Full comparative analyses calibrated for the United Kingdom and Japan are provided in S1 Appendix to corroborate that the identified feasibility gap is a universal phenomenon independent of a specific nation’s baseline capacity.

### Reconstruction of historical epidemics

#### Data extraction and standardization.

To provide an empirical benchmark for our predictive models, we reconstructed the daily hospital bed demand for the 1918 (H1N1), 1957 (H2N2), and 1968 (H3N2) influenza pandemics from weekly excess mortality estimates [19]. We note that this reconstruction does not require statistically fitting a model to empirical time-series data, but rather utilizes the empirical time-series data directly.

The 1918 pandemic propagated through distinct regional waves in the US, rather than as a simultaneous national event. We selected the US West Coast wave to serve as a representative morphological baseline since this regional wave exhibited an intermediate intensity profile, more acute than the Midwest but less peaked than the East Coast. We normalized the West Coast data to reconstruct the results as if a wave of that same regional intensity had struck the entire U.S. simultaneously. See S1 Appendix for details. Importantly, these historical reconstructions serve strictly as epidemiologic, not therapeutic, analogues. Because modern interventions like antivirals were unavailable in 1918, we use these historical incidence curves purely as a baseline transmission layer upon which modern pharmaceutical mitigation is theoretically superimposed.

#### Transformation to hospital demand.

We estimated continuous daily mortality curves using Piecewise Cubic Hermite Interpolating Polynomial (PCHIP) interpolation. These were converted into hospital bed demand using a mortality-to-admission back-calculation that assumes a 10% in-hospital mortality rate and a uniform 4-day length of stay (LoS), see S1 Appendix for transformation equations. While a 4-day LoS is consistent with observations of moderate influenza infections, empirical data from previous pandemics and severe seasonal outbreaks suggest that this duration substantially underestimates the resource requirements for critically ill patients, who often require extended care [24–26]. By utilizing this conservative LoS, our model intentionally establishes a ‘best-case’ demand floor to test the fundamental limits of healthcare capacity.

We explicitly acknowledge the inherent limitations of this transformation. Care delivery, clinical thresholds for admission, and therapeutic capabilities in 1918, 1957, and 1968 differ fundamentally from modern standards. Consequently, this back-calculation does not attempt to reconstruct historical hospital occupancy. Instead, it utilizes historical epidemic curves purely to capture the empirical morphology of real-world transmission waves. By applying modern, highly conservative clinical parameters to these established data, we generate a synthetic modern stress test rather than a direct historical clinical analogue.

#### Predictive modeling of planning scenarios.

Distinct from the historical reconstruction, we developed a forward-looking modeling framework to simulate hypothetical future pandemics based on official government planning assumptions.

- **Scenario parameters:** We adopted the United States Centers for Disease Control and Prevention (CDC) Pandemic Severity Assessment Framework [7] to define Moderate, Severe, and Very Severe parameters. These scenarios specify clinical attack rates (20% to 30%) and hospitalization rates (1.25%–12% of symptomatic cases). Comparable parameters were derived for the UK and Japan using their respective national preparedness strategies [6,23], see Tables A and B in S1 Appendix for parameter tables.
- **Static estimation (theoretical lower bound):** We first established a conservative baseline using a static calculation that assumes a flat, uniform epidemic curve over a given duration, e.g., six weeks. This method distributes the total caseload evenly, estimating a constant daily bed demand independent of transmission dynamics. We utilize this biologically unrealistic distribution as a best-case argument to define the absolute minimum stress on the healthcare system; if the stockpile strategy fails under these optimized conditions, it will inevitably fail under realistic transmission dynamics, see S1 Appendix for details.
- **Dynamic SIHR modeling:** To capture the acute surge dynamics masked by the static model, we employed a deterministic Susceptible-Infectious-Hospitalized-Recovered (SIHR) model (see Eq. 1 in S1 Appendix). We assume uniform population mixing, where demographic and social contact structures are mathematically absorbed into an effective basic reproduction number (R0). The model was calibrated to reproduce the specific wave durations (e.g., 6, 8 and 15 weeks in the US, Japan and UK scenarios, respectively) and cumulative attack rates defined in the various scenarios using a multi-step algorithm:
  1. **Timing:** The transmission rate (*β*) was tuned to constrain the epidemic duration to the desired duration, i.e., according to the planning scenario.
  2. **Magnitude:** The hospitalization probability (*p*) was adjusted to ensure the model’s projected volume of admissions aligned with the target clinical attack rate, see S1 Appendix for calibration algorithm.

A sensitivity analysis was conducted to confirm the robustness of results to variations in the infectious period, which influences the generation time, see Fig D in S1 Appendix. The other parameters act as simple scalar multipliers on the hospital load. For example, a 10% increase in either the clinical attack rate or the LoS results in a proportional 10% increase in peak bed demand.

#### Healthcare capacity thresholds.

To define system saturation, we established Stress Zones based on empirical observations of US healthcare performance degradation during COVID-19 [27]:

- **Severe strain:** 50,000–100,000 additional daily patients.
- **Crisis zone:** 100,000–150,000 additional daily patients.
- **Systemic collapse:** >150,000 additional daily patients.

For the comparative case studies of the United Kingdom and Japan, we evaluated saturation by directly comparing projected demand against the limit of available staffed beds, see S1 Appendix for country-specific capacity data and model parameters.

#### Intervention modeling.

We modeled the impact of antiviral intervention (neuraminidase inhibitors) solely within the predictive framework. We assumed a theoretical unlimited stockpile scenario where treatment is provided to 100% of symptomatic cases, explicitly neglecting logistical constraints. While these uniform assumptions do not capture real-world operational frictions, such as delayed administration, they are intentionally designed to stress-test the absolute limits of healthcare capacity under idealized conditions. Antiviral effectiveness was parameterized as a 30%–60% reduction in hospitalization risk, representing a range of optimistic clinical outcomes derived from major meta-analyses of oseltamivir efficacy [28–31]. We compared the daily bed demand in these mitigated (best-case) versus unmitigated scenarios against the defined capacity thresholds, see S1 Appendix for intervention modeling equations.

To evaluate the role of NPIs, we modeled NPIs as a reduction in the effective transmission rate (*β*) sufficient to lower the effective reproduction number (Rt) to a target level (e.g., Rt = 1.12) that maintains hospital demand within determined capacity limits, see S1 Appendix for details.

#### Uncertainty quantification.

To clarify the scope of our uncertainty analysis: Quantified uncertainties include the variation in our five primary epidemiological parameters (evaluated via parameter sweeps in the sensitivity analysis; see Table B in S1 Appendix), the clinical efficacy of antivirals (modeled as a range from 30% to 60%), and the baseline transmission severity (evaluated across Moderate, Severe, and Very Severe planning scenarios). Unquantified uncertainties relate primarily to our parsimonious model structure and operational data abstractions. Specifically, to establish the theoretical upper bounds of stockpile effectiveness, we deliberately did not quantify the uncertainties arising from heterogeneous population mixing, logistical distribution delays (assuming frictionless 100% coverage), or granular sub-system bottlenecks (e.g., shortages of mechanical ventilators or specialized staff, as opposed to general bed capacity).

### Artificial intelligence disclosure

During the preparation and revision of this manuscript, the authors utilized Google Gemini 3.1 exclusively to assist with language editing, grammatical refinement, and structural formatting of the manuscript text. No artificial intelligence tools were used in the conceptualization of the study, the formulation of the SIHR models, the data analysis, or the interpretation of the clinical and historical findings. The authors retain full and final responsibility for the entirety of the manuscript, including all findings, data, and supporting materials.

## Results

### Highly severe pandemic scenarios: Systemic collapse

Our analysis indicates that stockpile-based planning is fundamentally insufficient to prevent healthcare system collapse during high-severity pandemics, as illustrated by the Very Severe scenario (CDC planning parameter: mid-range 25% clinical attack rate, 12% hospitalization rate) in Fig 1. Even under the conservative static lower-bound estimation shown in Fig 1A, which assumes a flat, uniform distribution of cases over 6 weeks, the unmitigated daily demand exceeds 900,000 beds. Implementation of a fully optimized antiviral stockpile treatment (30%–60% effectiveness) reduces this peak to a prevalence of approximately 500,000 beds, a level that still exceeds the US national surge capacity by more than 3-fold [32]. When accounting for a more realistic surge dynamics using the transmission model in Fig 1B, the projected failure becomes absolute; the unmitigated peak demand surges to approximately 2 million daily beds. Even with a fully optimized treatment using unlimited antiviral stockpile (100% coverage of symptomatic infections; 30%–60% antiviral effectiveness, the mitigated peak would still range between 800,000 and 1.4 million beds. These levels exceed the national surge capacity by a factor of roughly 5–9, rendering the stockpile structurally insufficient for preventing system collapse. A standardized historical reconstruction of the 1918 pandemic, presented in Fig 1C, corroborates this scale of failure, yielding a dynamic peak well into the millions where a theoretical massive antiviral intervention would only reduce the peak to levels that remain well above the healthcare system’s collapse threshold.

**Fig 1:**
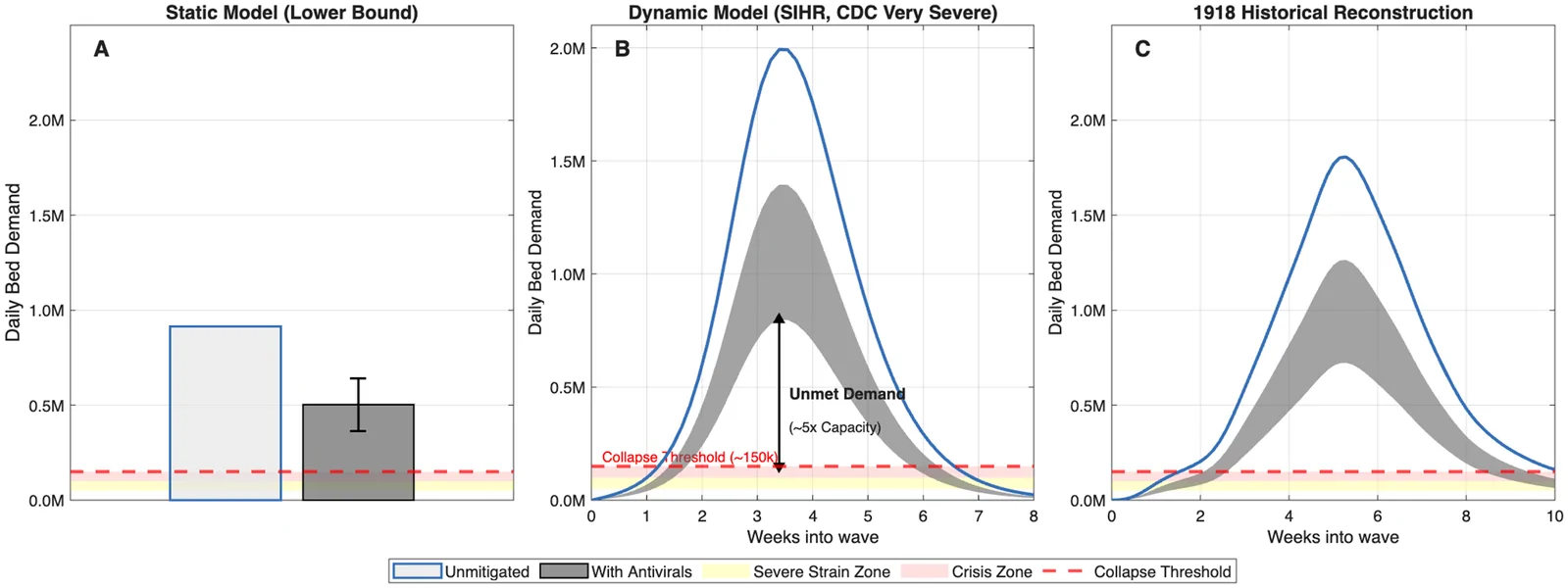
Projected daily hospital bed demand under Very Severe pandemic scenarios. **(A)** Static lower-bound estimate assuming a uniform distribution of cases over 6 weeks. **(B)** Dynamic transmission model calibrated to CDC Very Severe planning parameters (mid-range 25% clinical attack rate; 12% hospitalization rate). **(C)** Historical reconstruction of the 1918 pandemic (US West Coast incidence data) standardized to the 2015 US population. This reconstruction serves strictly as an epidemiological baseline for transmission dynamics, onto which modern pharmaceutical mitigation is theoretically superimposed (see Methods for details). Note that in the reconstruction (C) the pandemic’s duration is about 8 weeks while in the simulation (B) it is only 6 weeks blue outlines represent projected peak demand without pharmaceutical intervention. The dark gray shaded region represents the mitigated bed demand under 100% antiviral coverage for symptomatic cases, spanning the 30%–60% range of treatment-induced reduction. Error bars (panel A) indicate the upper and lower bounds of this reduction based on a 30%–60% efficacy range. Background colors denote healthcare system stress levels based on national bed capacity: Severe Strain Zone (yellow; 50,000–100,000 daily beds), Crisis Zone (pink; 100,000–150,000 daily beds), and Severely Exceeded Capacity (red; >150,000 additional daily beds). Note that in all three models, mitigated demand exceeds the system’s collapse threshold by a factor of 3–9. Abbreviations: CDC, Centers for Disease Control and Prevention; US, United States.

### Severe scenarios: Persistent exceeded capacity

In the Severe scenario (CDC planning parameter: mid-range 25% clinical attack rate, 6% hospitalization rate), the results remain structurally identical, consistently exceeding the collapse threshold (>150,000 beds) as shown in Fig 2. The conservative static model in Fig 2A projects a daily peak demand of 460,000 hospital beds; theoretical maximum pharmaceutical mitigation reduces this to 180,000–250,000 beds, a residual volume that still exceeds the US national surge capacity [32]. Assuming a more realistic dynamics by using transmission modeling (Fig 2B) projects an even higher unmitigated peak of approximately 1 million beds. Optimal antiviral intervention potentially reduces this peak to a range of 400,000–700,000 beds, leaving the system in a state of total collapse with demand exceeding capacity by a factor of 3–5. This pattern of saturation is consistent globally; in the United Kingdom and Japan, mitigated peak demands in comparable scenarios similarly exceed national surge capacities, see S1 Appendix for details.

**Fig 2:**
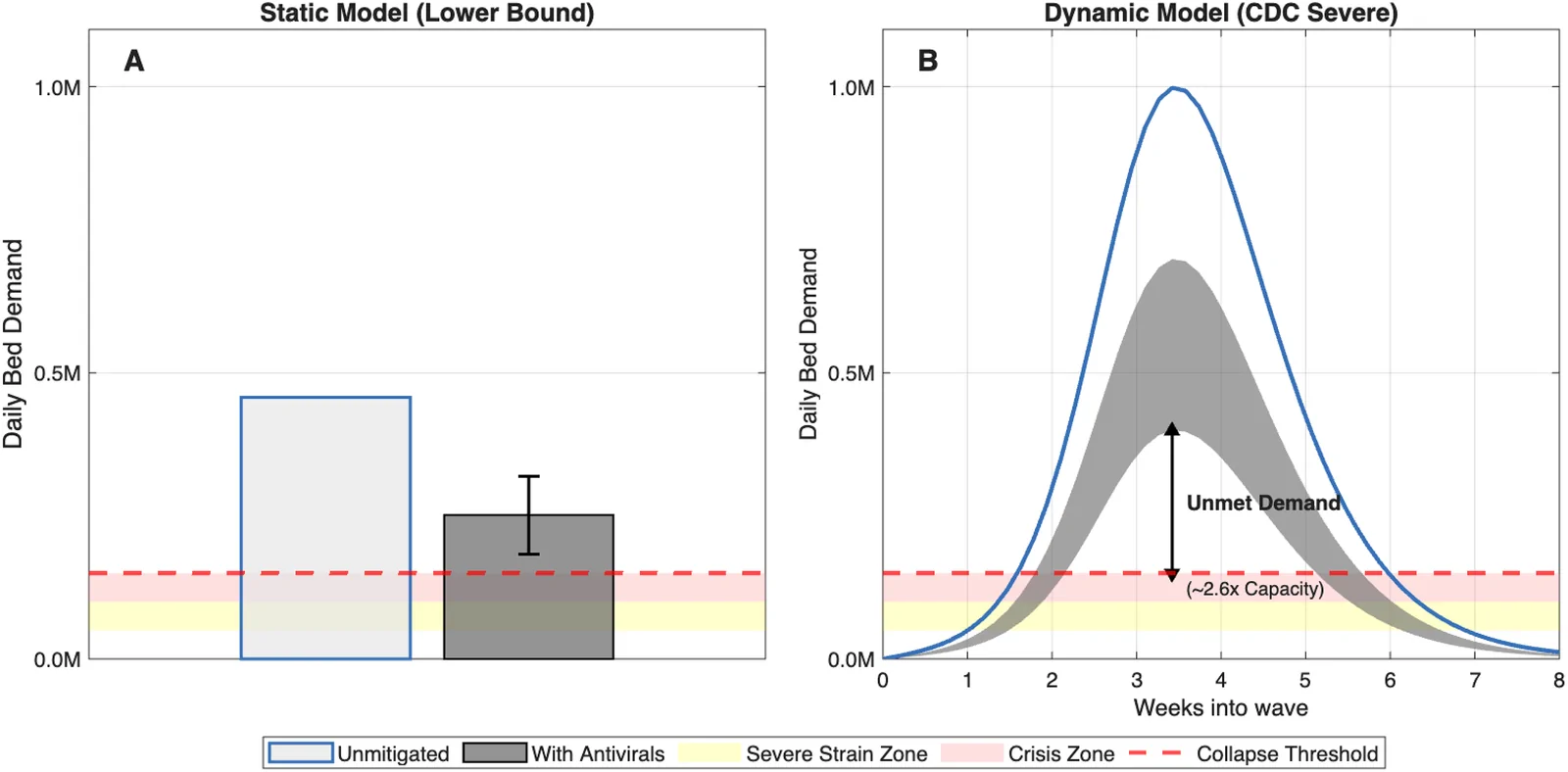
Projected daily hospital bed demand under Severe pandemic scenarios. **(A)** Static lower-bound estimate assuming a uniform distribution of cases over 6 weeks (see Methods for details). **(B)** Dynamic transmission model (see Methods and S1 Appendix) calibrated to CDC Severe planning parameters (mid-range 25% clinical attack rate; 6% hospitalization rate). The dark gray shaded region represents the mitigated bed demand under 100% antiviral coverage for symptomatic cases, spanning the 30%–60% range of treatment-induced reduction. Error bars indicate the upper and lower bounds of this reduction based on a 30%–60% efficacy range. Background colors denote healthcare system stress levels based on national bed capacity: Severe Strain Zone (yellow; 50,000–100,000 daily beds), Crisis Zone (pink; 100,000–150,000 daily beds), and Severely Exceeded Capacity (red; >150,000 additional daily beds). Note that in both models, mitigated demand exceeds the system’s collapse threshold by a factor of 2.6 to 4.6. Abbreviations: CDC, Centers for Disease Control and Prevention; US, United States.

### Stockpile viability in moderate scenarios

In contrast to very severe and severe events, antiviral stockpiles demonstrated considerable utility in moderate scenarios, as detailed in Fig 3. Under the CDC Moderate parameters (25% clinical attack rate,1.25% hospitalization rate), the unmitigated baseline demand in the static model (Fig 3A) is ~100,000 and in the dynamic model (Fig 3B) is ~210,000 beds, possibly exceeding the collapse threshold at 150,000 beds. Antiviral intervention successfully suppresses this peak to 50,000 for the static model and 150,000 beds in the dynamic model, keeping the system within the safety zone. Standardized historical reconstructions of the 1957 (Fig 3C) and 1968 (Fig 3D) pandemics further corroborate that for events of this magnitude, widespread treatment potentially can reduce the load to between 60,000 and 100,000 beds or lower, thereby preventing hospital bed exhaustion and ensuring the system remains functional.

**Fig 3:**
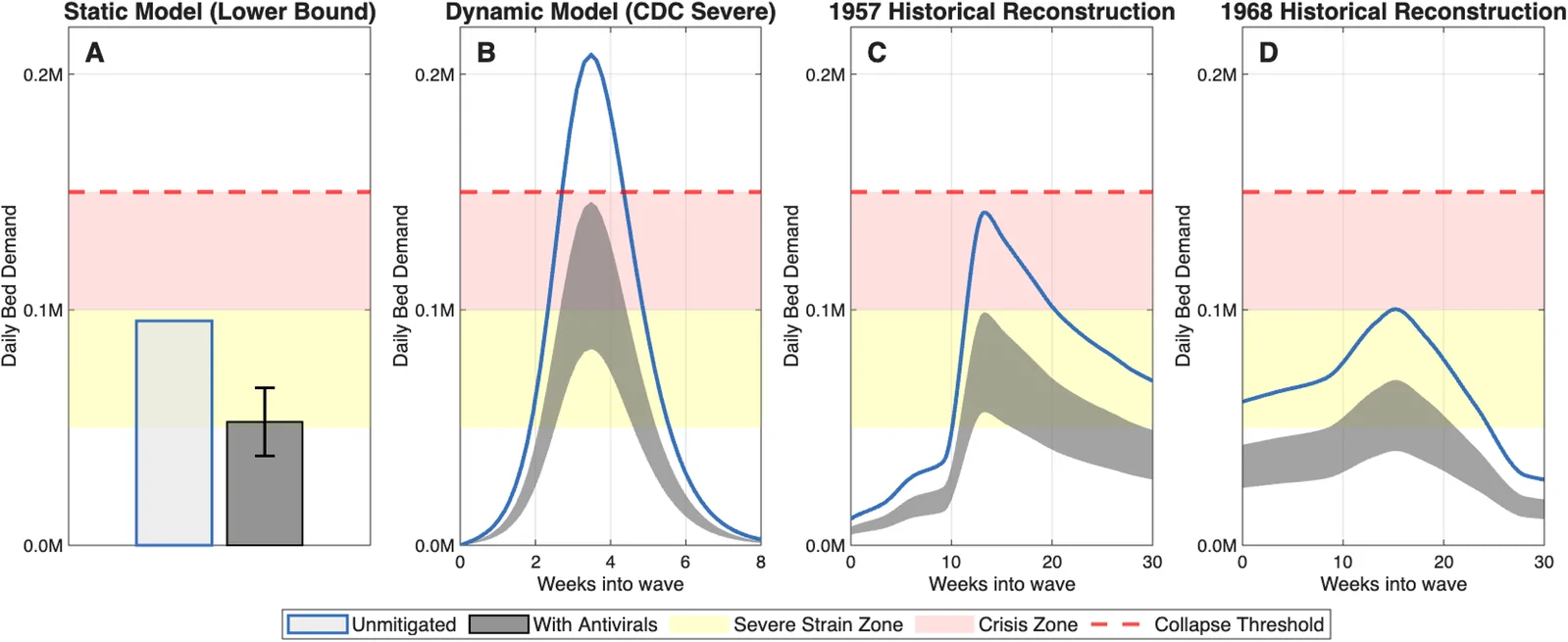
Projected daily hospital bed demand under Moderate pandemic scenarios. **(A)** Static lower-bound estimate assuming a uniform distribution of cases over 6 weeks. **(B)** Dynamic transmission model calibrated to CDC Moderate planning parameters (i.e., mid-range 25% clinical attack rate; 1.25% hospitalization rate). **(C)** Historical reconstruction of the 1957 pandemic standardized to the 2015 US population. **(D)** Historical reconstruction of the 1968 pandemic standardized to the 2015 US population. These reconstructions serve strictly as an epidemiological baseline for transmission dynamics, onto which modern pharmaceutical mitigation is theoretically superimposed. For full details regarding the models, see Methods and S1 Appendix. Blue outlines represent projected peak demand without pharmaceutical intervention. The dark gray shaded region represents the mitigated bed demand under 100% antiviral coverage for symptomatic cases, spanning the 30%–60% range of treatment-induced reduction, mitigated demand. Error bars indicate the upper and lower bounds of this reduction based on a 30%–60% efficacy range. Background colors denote healthcare system stress levels based on national bed capacity: Severe Strain Zone (yellow; 50,000–100,000 daily beds), Crisis Zone (pink; 100,000–150,000 daily beds), and Severely Exceeded Capacity (red; >150,000 additional daily beds). Abbreviations: CDC, Centers for Disease Control and Prevention; US, United States.

### Impact of non-pharmaceutical interventions

Given the limited capacity of antiviral stockpiles to avert system overload, we evaluated NPIs calibrated to reduce the effective reproduction number (Rt) from 1.56 to 1.12, see S1 Appendix. While highly stringent NPIs could suppress Rt below 1, such measures are often economically and societally unsustainable over long periods. Consequently, we calibrated Rt to 1.12 to simulate a more realistic, yet resource-intensive, mitigation strategy. This approach focuses on implementing the minimum level of intervention necessary to flatten the curve, aiming to keep peak hospital demand just below the system’s collapse threshold. However, maintaining this delicate balance over an extended duration places a substational and prolonged strain on public health resources and infrastructure. This intervention fundamentally alters the epidemic profile in three critical ways as shown in Fig 4. First, it suppresses the peak daily hospitalizations from approximately 400,000–700,000 to approximately 80,000–145,000, within the Severe Strain and Crisis zones and just below the capacity threshold (~150,000), preventing the surge from exceeding it. Second, the intervention markedly elongates the wave: the peak shifts from about week 3.5 to about week 6.5, and the wave extends beyond week 11. This flattens the curve by distributing the patient load over a much longer period rather than allowing a localized peak to overwhelm surge capacity. Third, by lowering the force of infection, the NPI strategy reduces the total clinical attack rate from the 25% baseline to approximately 8%, substantially limiting the total volume of patients requiring antivirals.

**Fig 4:**
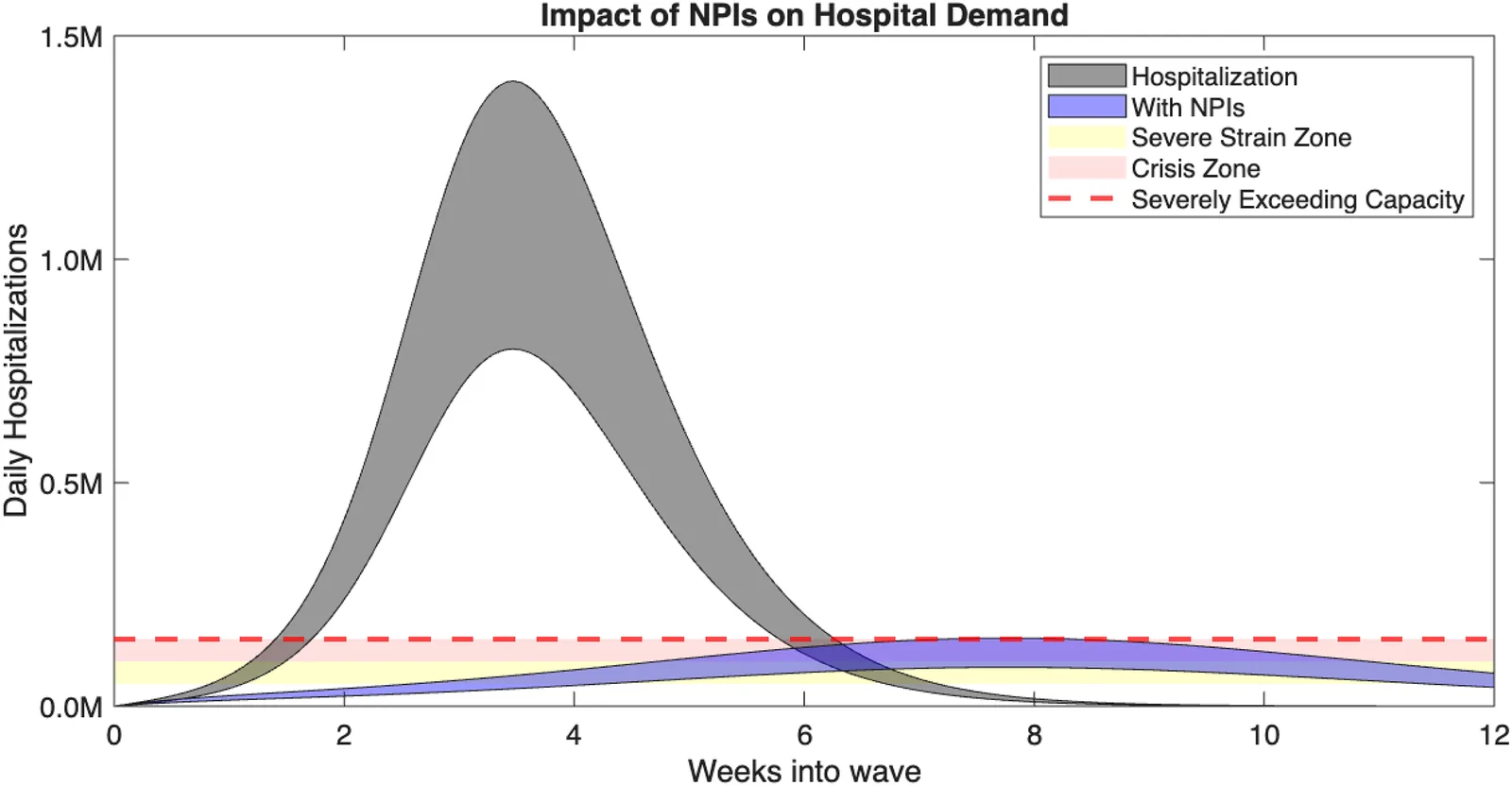
Impact of Non-Pharmaceutical Interventions (NPIs) on Hospital Demand. The dark shaded region represents the projected daily hospitalizations for the CDC Severe scenario using the transmission model with hospitalization rates of 6%. Given full treatment with antivirals provided to all infected individuals with an effectiveness of 30%–60%, but without Non-Pharmaceutical Interventions. The blue shaded region illustrates the mitigated demand trajectory following the implementation of NPIs so that the effective reproduction number is reduced to Rt = 1.12, in such a case only 8% of the population requires antivirals. **Capacity Zones:** Background colors denote healthcare system stress levels based on national bed capacity: Severe Strain Zone (yellow; 50,000–100,000 daily beds), and Crisis Zone (pink; 100,000–150,000 daily beds). The collapse threshold is marked by a red dashed line. Abbreviations: CDC, Centers for Disease Control and Prevention; US, United States.; NPIs, Non-Pharmaceutical Interventions; $R_{t}$, effective reproduction number.

### Robustness and sensitivity

To ensure our conclusions regarding systemic collapse are robust to parameter uncertainties, we conducted comprehensive sensitivity analyses (detailed in S1 Appendix). Because our calibration algorithm temporally anchors the epidemic wave to durations (e.g., 6, 8 and 15 weeks for US, Japan and UK respectively) defined by official planning scenarios, the projected capacity deficits remain remarkably stable against varying assumptions. These analyses confirm that reasonable parametric shifts (see Table C in S1 Appendix) in the five model parameters do not alter the fundamental conclusion: under severe scenarios, with treatment of unlimited antivirals without NPIs the healthcare system collapses.

## Discussion

Re-evaluating current policy frameworks is essential, as pandemic preparedness is inherently an exercise in managing uncertainty; the timing, severity, and viral characteristics of the next global health crisis remain unknown [1,2]. Consequently, strategic planning must be robust enough to withstand a wide spectrum of potential threats, ranging from moderate pandemics (e.g., 1957 or 1968) to catastrophic events comparable to 1918. Current strategies in high-resource nations, exemplified by the US, the UK, and Japan, rely heavily on the inventory-based presumption that pre-positioned stockpiles of neuraminidase inhibitors will be sufficient to mitigate these diverse threats [4–8]. However, this presumption rests on a fragile foundation. Our analysis demonstrates that these strategies fail to account for the rigid physical constraints of the healthcare systems they are meant to protect [15–17]. This structural oversight, compounded by the biological reality that static stockpiles may possess negligible efficacy against a novel strain [28–31], fundamentally challenges the validity of the current policy framework.

This feasibility gap creates a fundamental question regarding the return on investment for static reserves. Our results indicate that large stockpiles are potentially highly effective in moderate scenarios (e.g., 1957 or 1968 analogies), successfully suppressing peak demand from critical levels back into manageable territory. However, this reveals a difficult truth: antiviral stockpiles as a standalone policy are functionally most useful when the threat is inherently manageable. Conversely, in the severe scenarios where the system faces existential collapse, the stockpile is insufficient to avert disaster. This raises a fundamental question in resource allocation: Is the vast capital investment required to maintain massive, aging stockpiles justified if their primary utility is limited to scenarios that do not threaten the structural integrity of the healthcare system?

When pharmaceutical defenses are overwhelmed, public health authorities must deploy NPIs as a critical measure of last resort. While these interventions carry profound economic and societal costs [33], they act as a necessary brake against systemic disintegration by aligning patient demand with inelastic healthcare capacity. Because NPIs are inherently temporary, with transmission typically rebounding upon their removal, their primary strategic value lies in buying time rather than achieving permanent suppression.

Integrating NPIs with medical countermeasures restores the strategic utility of a national stockpile, even in severe pandemic scenarios. This synergy transforms the crisis landscape by elongating the epidemic wave and providing a critical response window to verify antiviral efficacy, while simultaneously lowering the overall attack rate to enable a potential smaller, more streamlined, and capacity-aligned stockpile. This framework offers a critical opportunity to revisit historical stockpiling paradigms and implement a capacity-based approach. By moving past cumulative metrics, combined with technological developments achieved over the last two decades since the traditional 25% population coverage benchmark was originally suggested. Furthermore, because the rapid advancement of messenger RNA (mRNA) vaccine platforms [34,35] has drastically compressed development timelines, this integrated approach allows considering a downsized antiviral reserve to transition from an insufficient standalone defense into a vital temporal bridge, maintaining systemic stability until ultimate vaccine deployment.

When interpreting these results, several inherent methodological limitations must be acknowledged. Our analysis utilizes a parsimonious, frictionless framework to establish the theoretical upper bounds of antiviral stockpiling effectiveness. By assuming perfect, instantaneous distribution to 100% of symptomatic cases and omitting logistical constraints or demographic targeting, the model carries an inherent optimistic bias. Consequently, the mitigating impact of antivirals is overstated, while the modeled NPI stringency (Rt = 1.12) likely underestimates the interventions required to prevent systemic collapse in practice.

This optimism is further compounded by three key assumptions. Firstly, understated healthcare demand; the projection of hospital demand relies on a conservative 4-day LoS that excludes the extended intensive care typical of severe respiratory infections, meaning healthcare saturation would occur more rapidly than projected. Secondly, capacity overestimation; by using general hospital beds as a proxy for capacity, the model abstracts away from critical sub-system bottlenecks, such as shortages of mechanical ventilators [36], extracorporeal membrane oxygenation (ECMO) machines, or specialized intensive care staff, which often precipitate systemic failure well before total bed capacity is exhausted. Finally, uncertain antiviral efficacy; in practice, the specific characteristics of a future viral strain may render existing stockpiles partially or entirely ineffective due to inherent or acquired resistance [37].

Furthermore, this parsimonious framework does not account for complex, targeted operational strategies used in a live pandemic response, such as post-exposure prophylaxis, the preservation of essential public services, or tactical bridging until vaccine deployment.

Nevertheless, these simplifications serve a vital analytical purpose. By demonstrating that a 25% standalone stockpile is mathematically insufficient to avert systemic collapse even under these best-case, frictionless assumptions, the model highlights a fundamental vulnerability in current policy paradigms that would only be magnified in a real-world crisis.

Our models establish the mathematical necessity of a capacity-based approach. The baseline definition of this approach is a policy whose primary goal is to maintain healthcare system integrity, which requires a fundamental shift away from relying solely on static, inventory-based stockpiles. A foundational principle central to operationalizing this shift is the strategic utilization of the pre-surge time window. It is important that pandemic scenarios account for temporal dynamics, specifically the expected time from initial cases to the emergence of a localized surge. Experience from past pandemics demonstrates a notable lag of several weeks from first notices to a full-scale surge [1,3,18]. Rather than a period of passive observation, this lag presents a crucial opportunity for gathering rapid epidemiological intelligence, evaluating the clinical efficacy of available antivirals against the novel pathogen, and establishing precise clinical guidelines [38]. Concurrently, it is essential that this window serves as a catalyst for operational readiness protocols: pre-positioning resources, shifting hospital operations, and distributing preliminary medical countermeasures before the surge fully materializes.

Operationalizing this capacity-based framework involves harmonizing proven interventions, such as NPIs and considering smaller targeted anti-viral reserves, with exploratory strategies that warrant further investigation. Potential additions to this arsenal include decentralized home-first care models [39], smartwatch-based health monitoring [40,41], and dynamic warm-base manufacturing. Translating these mathematical insights into actionable policy necessitates multi-disciplinary research that accounts for specific logistical constraints, healthcare capacities and regional variations.

In conclusion, the historical 25% antiviral stockpile mandate reflects a legacy planning era that viewed pandemics primarily through the lens of cumulative disease burden rather than acute operational threats. Our analysis confirms that while large reserves are effective for moderate outbreaks, they are fundamentally insufficient as a standalone measure to prevent healthcare collapse in high-severity scenarios, even under optimistic assumptions of perfect distribution and clinical efficacy.

As a substantial portion of current global antiviral stockpiles nears expiration, policymakers are presented with a critical inflection point. Acknowledging the physical limitations of an exclusively inventory-focused mindset provides a timely opportunity to correct a strategic blind spot and formally transition toward the resilient, capacity-preserving systems outlined above [42].

To support this shift, it is important that the scientific community remains engaged in developing dynamic modeling techniques to recalibrate the scale and deployment of future reserves, ensuring they function as a strategic bridge rather than a sole line of defense. This shift provides a vital opportunity to directly revisit the fundamental question of stockpile size, moving past legacy percentages toward a modern framework tailored to actual healthcare capacity. Future research should expand upon this baseline by integrating additional real-world factors that are critical to shaping future stockpiling policies, such as the logistics of rapid supply chain distribution, demographic targeting of high-risk populations, shifting patterns of public adherence to non-pharmaceutical interventions, and the economic sustainability of long-term reserve maintenance. Ultimately, we hope this work will catalyze a comprehensive reevaluation of pandemic preparedness [43,44], inspiring the design of adaptable, capacity-aligned strategies that are truly equipped to navigate the next global health crisis.

## Supporting information

S1 Appendix Supplementary figures and tables. (PDF)

## Abbreviations

CDC: Centers for Disease Control and Prevention
LoS: length of stay
NPIs: non-pharmaceutical interventions
PCHIP: Piecewise Cubic Hermite Interpolating Polynomial
SIHR: Susceptible-Infectious-Hospitalized-Recovered
