# Supplementary material for: Transforming pandemic influenza antiviral stockpiling using a capacity-based approach: A modeling study
Source: PLoS Med. 2026 Nov 3;23(11):e1004992. doi: 10.1371/journal.pmed.1004992 (PMC13730355; doi:10.1371/journal.pmed.1004992)
Supplement: Supplementary file 1 [file pmed.1004992.s001.pdf]

Appendix S1

Transforming Pandemic Influenza Antiviral Stockpiling: A Capacity-Based Approach

Nir Gavish and Amit Huppert

Mathematical Transmission Model

To evaluate the impact of infection dynamics on healthcare capacity, we employed a deterministic compartmental model extended from the classical SIR framework. The population N is subdivided into four compartments: Susceptible (S), Infectious in the community (I), Hospitalized (H), and Recovered (R). The model relies on three core assumptions: (1) **Homogeneous Mixing** within a closed population; (2) **Uniform Contribution to Transmission**, assuming that disease severity does not alter infectivity and that individuals destined for hospitalization generate the same force of infection while in the community as those with mild infections; (3) **Negligible demographic changes** (births/deaths) given the short epidemic horizon and (4) **Perfect distribution of antiviral treatment** so that, if antivirals are used, every symptomatic case is treated with antivirals.

The dynamics are depicted in Diagram A and governed by the following system of ordinary differential equations:

$$\begin{aligned}
\frac{dS}{dt} &= -\frac{\beta S(t)I(t)}{N} \\
\frac{dI}{dt} &= \frac{\beta S(t)I(t)}{N} - \gamma_I I(t) \\
\frac{dH}{dt} &= (1-\varepsilon)p \cdot \gamma_I I(t) - \gamma_H H(t) \\
\frac{dR}{dt} &= (1-(1-\varepsilon)p)\gamma_I I(t) + \gamma_H H(t)
\end{aligned} \tag{1}$$

where β is the effective transmission rate and γ_I is the removal rate from the community, so that the average infectious period is $1/\gamma_I$. The parameter p represents the probability that an infected individual requires hospitalization. Individuals leave the hospital compartment at rate γ_H, so that the average length of stay is $1/\gamma_H$. Antiviral efficiency in preventing hospitalization is given by ε. The total population $N = S+I+H+R$ remains constant. The basic reproduction number, R_0, representing the expected number of secondary cases produced by a single typical infection in a completely susceptible population, is defined as $R_0 = \beta/\gamma_I$.

Effect of antiviral treatment

To model pharmaceutical intervention, we set the antiviral efficiency ε to be non-negative (0.3 to 0.6).

Modelling Non-Pharmaceutical Interventions (NPIs)

To evaluate the impact of Non-Pharmaceutical Interventions (e.g., social distancing, school closures, lockdowns) on healthcare capacity, we simulated a reduction in the effective transmission rate, β, so that a target effective basic reproduction number is achieved,

$$\beta_{NPI} = R_{target} \times \gamma_I. \tag{2}$$

For our examples, we manually chose R_{target} to a level that keeps hospital demand within manageable limits.

Estimation of Peak Hospitalization Demand

To estimate daily hospital demand for various planning scenarios using the SIHR model, we developed a four-step algorithm. Out of the five model parameters, the transmission rate β and hospitalization rate p are computed, while the three remaining parameters are pre-determined. Following [7], we set $\gamma_I = 0.5$ reflecting an average infectious

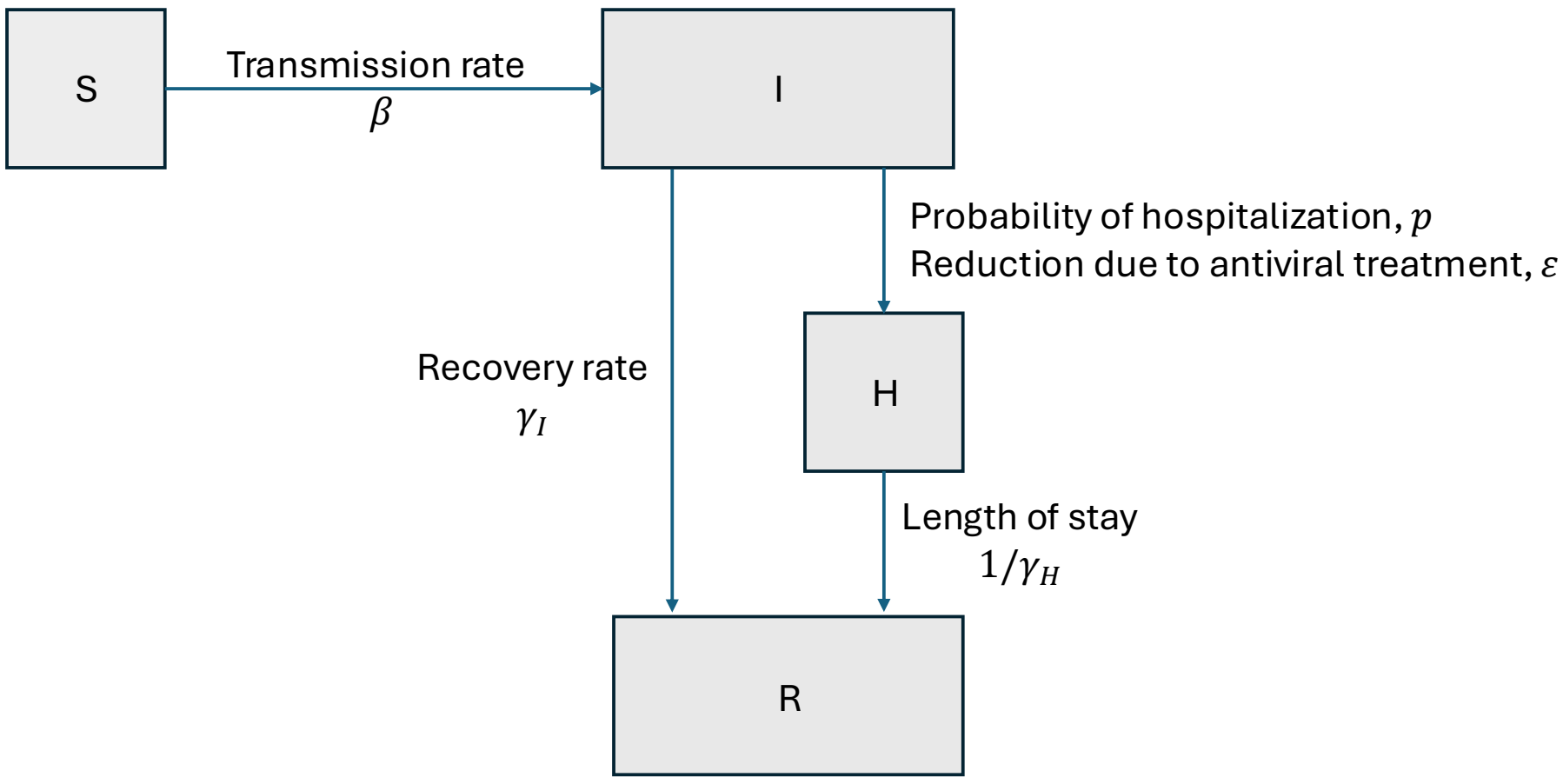

Figure A: **Structure of the deterministic SIHR compartmental model.** The diagram illustrates the disease natural history, the determinants of acquisition, and the integration of the putative antiviral intervention. Susceptible (S) individuals transition to the infected (I) compartment governed by the transmission rate (β). Infected individuals either progress directly to the recovered (R) compartment based on the recovery rate (γ_I) or develop severe disease requiring transition to the hospitalized (H) compartment. This transition is determined by the baseline probability of hospitalization (p) and is modified by the putative intervention (antiviral treatment, ε). Hospitalized patients eventually transition to the recovered compartment determined by their average length of stay ($1/\gamma_H$). *Abbreviations: SIHR, Susceptible-Infected-Hospitalized-Recovered.*

period of 2 days, and assume an hospitalization period of 4 days, $\gamma_H = 0.25$ [3, 10, 6]. The sensitivity of our results to these parameter choice is discussed subsequently.

The triple-step algorithm calibrates transmission dynamics against the duration and magnitude constraints defined in the considered pandemic planning framework.

Step 1. Calibration of Transmission Timing. We first determined the specific transmission rate, β, required to constrain the epidemic duration (e.g., ∼6 weeks) such that the peak prevalence of infection occurs in the mid-period (e.g., ∼3 weeks). This was achieved by solving the inverse problem using the root-finding algorithm (`fzero`) in Matlab for the equation

$$t_{\text{peak}}(\beta) = t_{\text{mid}},$$

where $t_{\text{peak}}(\beta)$ is the time at which $dI/dt = 0$, and t_{mid} is the mid-period time. To compute $t_{\text{peak}}(\beta)$, we solve the SIHR model with the initial condition set to 0.1% of the population $I(0) = 10^{-3}N$ until $I(t)$ reaches a peak. In this step, hospitalization is neglected, $p = 0$, since, by model assumptions, it has no effect on the epidemic duration.

Step 2. Adjustment of clinical attack rate. Using the value of β derived in Step 1, we calculated the projected total attack rate, Z, (cumulative incidence) for the epidemic

$$Z = \gamma_I \int_0^\infty I(t)dt.$$

We then adjusted the hospitalization probability parameter, p, to align the model's output with a target clinical attack rate

$$p = \frac{Z_{\text{clinical}}\, p_{\text{hospital}}}{Z}$$

where Z_{clinical} is the given clinical attack rate (e.g., 20-30%) and p_{hospital} is the fraction of those with symptoms (clinical attack rate) that require hospitalization.

Step 3. Determination of Dynamic Peak. The system of differential equations (1) was integrated using the calibrated parameters to identify the maximum value of the hospitalization curve:

$$H_{\text{peak}}^{\text{dynamic}} = \max_t H(t) \tag{3}$$

Comparison with Uniform Capacity Estimates.

We compared the dynamically computed peak, $H_{\text{peak}}^{\text{dynamic}}$, against a lower-bound estimate based on an over-simplified assumption of a flat epidemic curve. This estimate assumes that infections are distributed evenly over the epidemic window, e.g., over a period of six weeks. This static benchmark is calculated as:

$$H_{\text{peak}}^{\text{flat}} = \frac{N \times AR_{\text{clinical}} \times p_{\text{hospitalization}} \times D_H}{D_{\text{epidemic}}} \tag{4}$$

Where N is the total population size, AR_{clinical} is the clinical attack rate, $p_{\text{hospitalization}}$ is the proportion of clinical cases requiring hospitalization, D_H is the average duration of hospital stay and D_{epidemic} is the duration of the epidemic wave.

Comparative Analysis of Pandemic Planning

The following table summarizes the demographic baselines for the United States, United Kingdom, and Japan. We note that while we use free surge bed capacity as a comparative baseline, it may not be a good approximate for the collapse threshold. For example, while the free surge bed capacity in the USA is roughly 320,000 staffed bed, the experience in COVID-19 implies that the US health system reaches systemic collapse already at 150,000 additional daily patients [2].

Table A: Comparative Analysis of Pandemic Planning Parameters

**Parameter**	**United States** [1, 12]	**United Kingdom**[4, 9, 13, 11, 5]	**Japan** [8]
**I. Demographics & Capacity**			
Population	∼320 Million (in 2015)	∼69.5 Million	∼123.8 Million
Staffed Hospital Bed Count*	∼920,000	∼176,000	∼900,000
Baseline Occupancy*	∼65%	∼92–94%	∼80%
”Free” Surge Beds (Approx)	∼322,000	∼14,800	∼180,000

** Corresponds to the staffed bed count of 2024 without standardizing it to the population of the United States in 2015.*

Table B summarizes planning scenarios (”Reasonable Worst Case”) and model parameters. and

Table B: Calibrated Model Parameters by Scenario

	**Model Constants & Calibrated Values**				**Scenario Targets (Inputs)**		
**Scenario**	β	p	γ_I	γ_H	**Duration**	**Clinical AR**	**Hosp. Rate***
USA (Very Severe)	0.78	0.0483	0.5	0.25	6 Weeks	25%	12.0%
USA (Severe)	0.78	0.0242	0.5	0.25	6 Weeks	25%	6.0%
USA (Moderate)	0.78	0.0050	0.5	0.25	6 Weeks	25%	1.25%
United Kingdom	0.31	0.03	0.2**	0.25	15 Weeks	50%	4.0%
Japan	0.69	0.0400	0.5	0.25	8 Weeks	25%	7.8% ***

Abbreviations: USA, United States of America; AR, Attack rate.

** Hospitalization rate refers to the percentage of symptomatic/clinical cases requiring hospitalization, whereas p represents the effective probability of hospitalization per infection used in the SIHR model equations.*

***Note on UK's average infectious period: While our standard simulations set the recovery rate to $\gamma_I = 0.5$ to reflect an average infectious period of 2 days (following [7]), this assumption proved structurally incompatible with the extended duration of the UK scenario. Specifically, maintaining a short 2-day infectious period over such a prolonged wave yields an attack rate that falls significantly short of the target 50% clinical attack rate. To properly align the model's transmission dynamics with this higher attack rate over a longer temporal baseline, we adjusted the parameter to $\gamma_I = 0.2$, which corresponds to an extended average infectious period of 5 days.*

****Note on Japan's Hospitalization Rate: The Japanese government plans for specific patient totals rather than a fixed rate. In the "Severe" scenario, they a define a clinical attack rate of 25% resulting in admissions of 2.53 million. Standardizing to the Japanese demography at the time of report, implies a hospitalization rate of roughly 7.8%.*

Modelling Results

Figures B and C present the Projected daily hospital bed demand for national scenarios of the United Kingdom and Japan, respectively. Table C presents the comparative results of the dynamic SIHR modelling versus the uniform planning distribution for the three national scenarios.

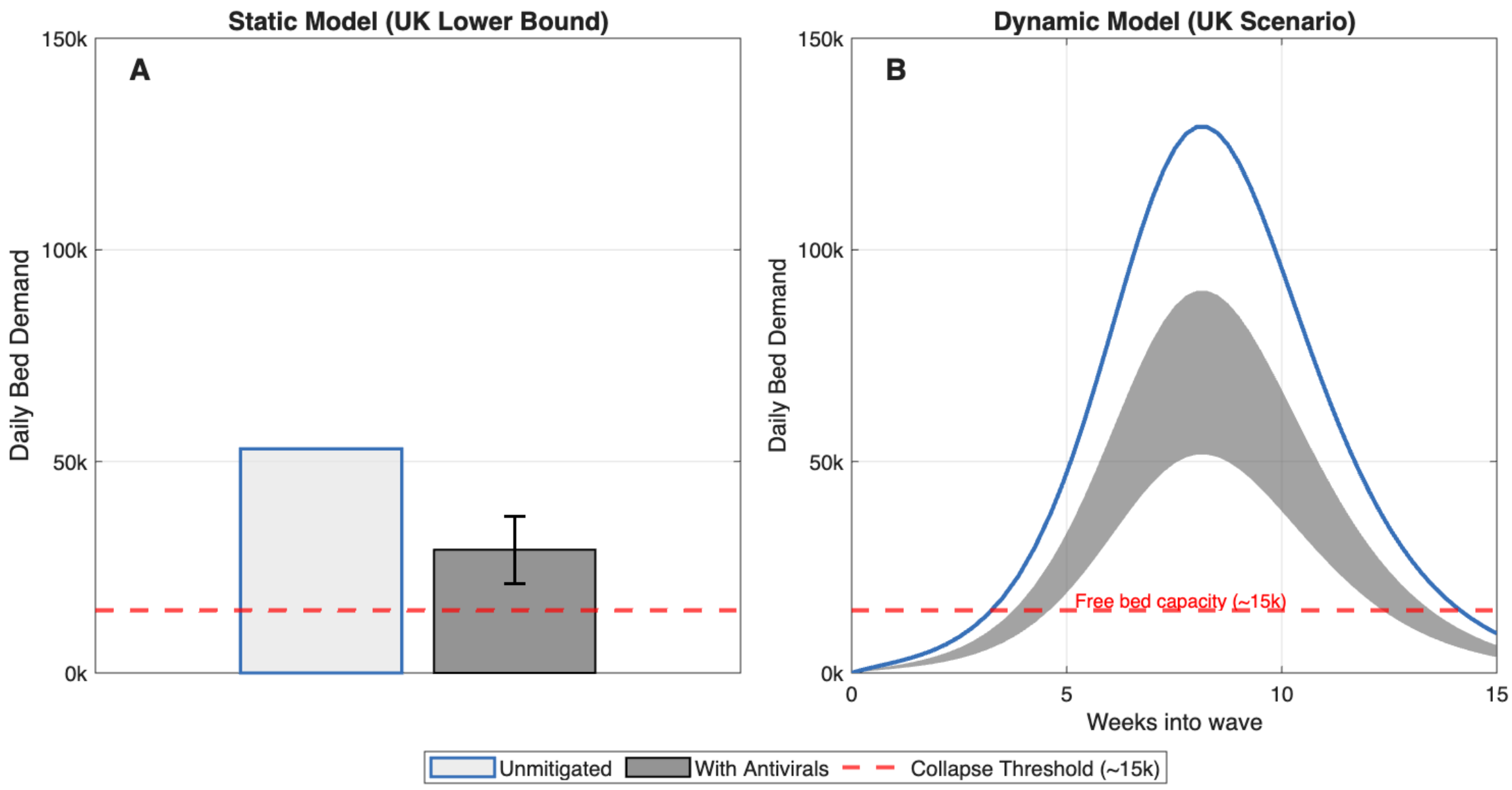

Figure B: Projected daily hospital bed demand under the United Kingdom pandemic scenario. (A) Static lower-bound estimate assuming a uniform distribution of cases over a 15-week wave. (B) Dynamic transmission model calibrated to UK planning parameters (50% clinical attack rate; 4.0% hospitalization rate). Dark grey shaded regions represent the reduced demand if antivirals were provided to 100% of symptomatic cases with an effectiveness of 30–60%. The red dashed line denotes the healthcare system's collapse threshold based on estimated free bed capacity (∼15,000 daily beds). Abbreviations: UK, United Kingdom.

Table C: Comparison of Static vs. Dynamic Peak Bed Demand Estimates

**Scenario / Region**	**Uniform Estimate** ($H_{\text{peak}}^{\text{flat}}$)	**Dynamic Peak** ($H_{\text{peak}}^{\text{dynamic}}$)	**With Antivirals (Mitigated; $\varepsilon = 0.3 - 0.6$)**	**"Free" Surge Beds (Approx)**
United States* (Moderate)	∼95,000	210,000	84,000 – 147,000	322,000
United States* (Severe)	460,000	1 Million	0.4 – 0.7 Million	322,000
United States* (Very Severe)	∼915,000	2 Million	0.8 – 1.4 Million	322,000
United Kingdom	∼53,000	129,000	51,000 – 77,000	14,800
Japan	∼172,000	426,000	170,000 – 256,000	180,000

* We use the mid-range clinical attack rate of 25%, and consider the range of hospitalization rates between 6-12% corresponding to the range of severe to very severe.

Sensitivity Analysis

We analyzed the sensitivity of peak capacity demand (H_{peak}) to key model parameters. The model structure implies that H_{peak} is linearly dependent on the population size (N), the clinical attack rate ($AR_{clinical}$), the hospitalization

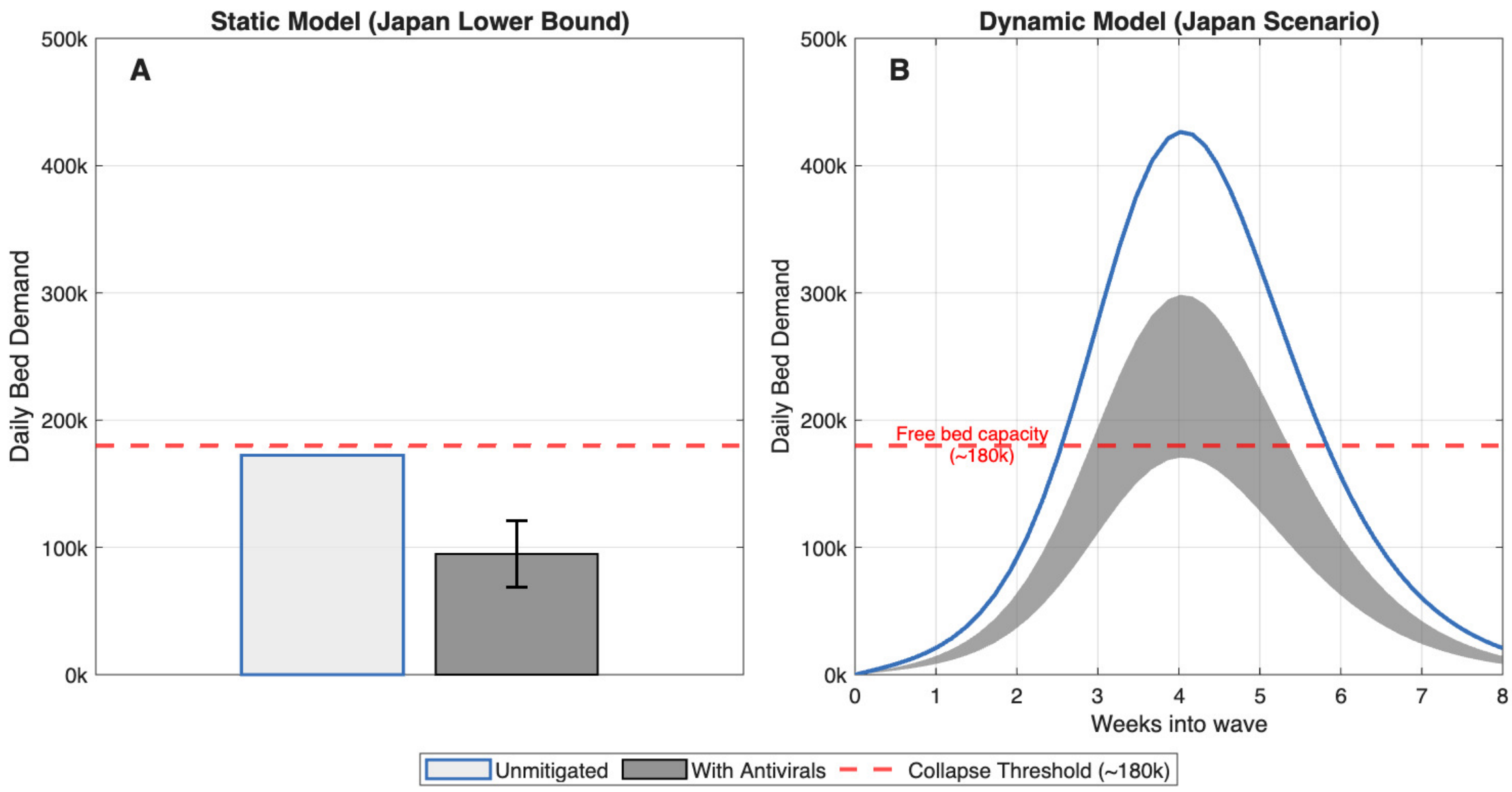

Figure C: Projected daily hospital bed demand under the Japan pandemic scenario. (A) Static lower-bound estimate assuming a uniform distribution of cases over an 8-week wave. (B) Dynamic transmission model calibrated to Japanese planning parameters (25% clinical attack rate; 7.8% hospitalization rate). Dark grey shaded regions represent the reduced demand if antivirals were provided to 100% of symptomatic cases with an effectiveness of 30–60%. The red dashed line denotes the healthcare system's collapse threshold based on estimated free bed capacity (∼180,000 daily beds).

probability (p), and the average hospital length of stay (D_H). Since the transmission dynamics (S and I compartments) determine the timing of the curve, these magnitude parameters act as simple scalar multipliers on the hospital load. For example, a 10% increase in either the clinical attack rate or the length of stay results in a proportional 10% increase in peak bed demand.

The infectious period ($D_I = 1/\gamma_I$), however, exhibits a non-linear relationship with peak demand (Figure D), as variations in this parameter fundamentally alter the basic reproduction number ($R_0 = \beta/\gamma_I$). To test the sensitivity of changes in γ_I, we vary γ_I and for each value of γ_I, we estimate the peak hospitalization demand using the triple-stage algorithm. In particular, β varies with γ_I. Our analysis reveals that the peak capacity demand is remarkably robust to this parameter. Across a wide range of infectious periods (1.5 to 10 days), the peak capacity demand varies only within the narrow range of 1.8 to 2.07 million staffed hospital beds. This confirms the robustness of our core findings.

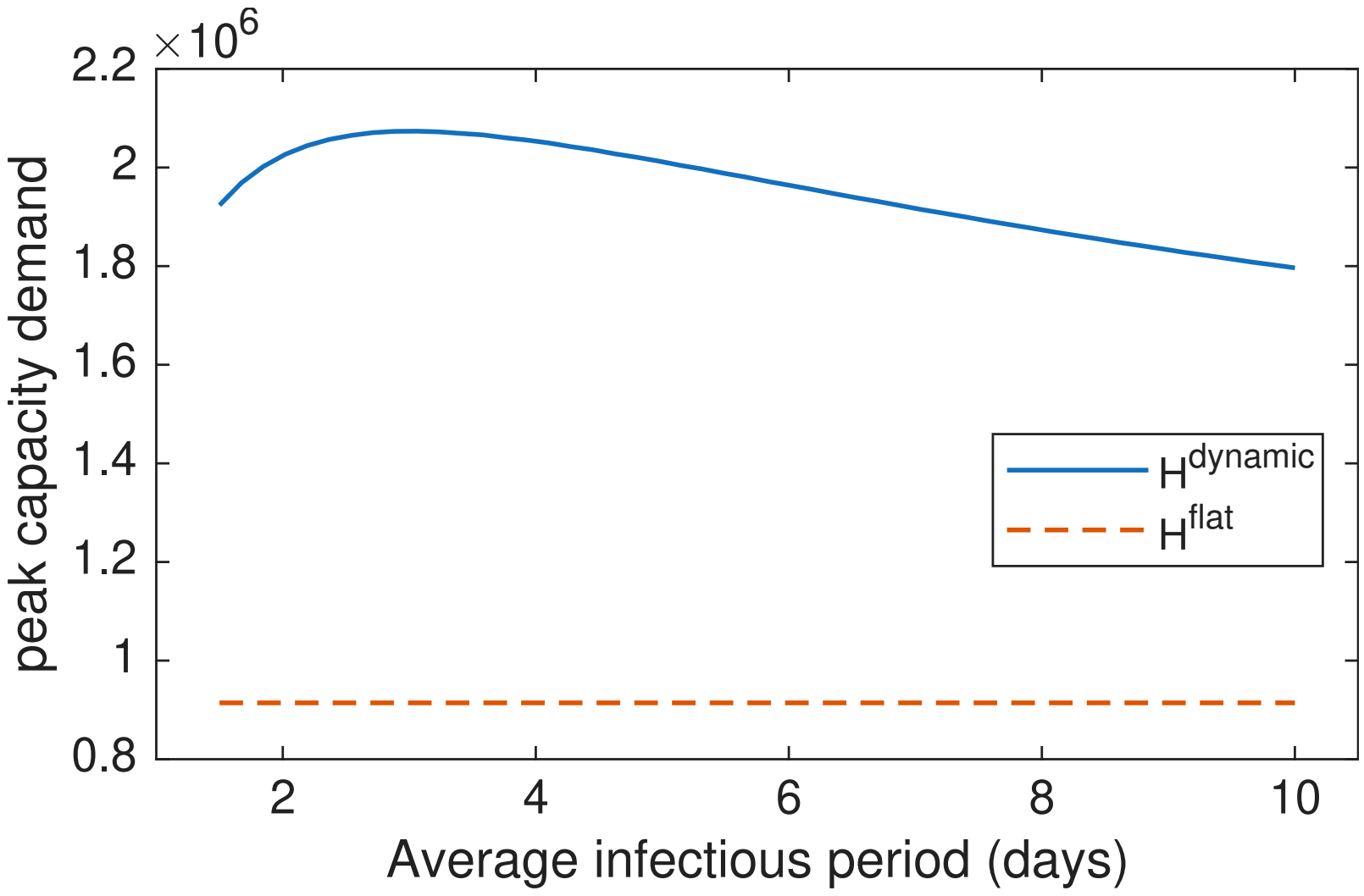

Figure D: Sensitivity of Peak Hospitalization Demand to Infectious Period. The solid blue curve illustrates the dynamic peak ($H_{\text{peak}}^{\text{dynamic}}$) as a function of the average infectious period ($1/\gamma_I$), while the dashed red line represents the static uniform capacity estimate ($H_{\text{peak}}^{\text{uniform}}$). The divergence highlights the non-linear impact of generation time on surge capacity.

The structural stability of the epidemiological model is fundamentally influenced by the calibration methodology. Because the wave peak is strictly anchored to the mid-period of the scenario's defined duration (as detailed in Table B), the overall results are significantly less sensitive to arbitrary parameter fluctuations. For example, in the USA scenarios which define a 6-week duration, the peak is explicitly anchored to day 21. By forcing the model to align with this fixed temporal milestone, the calibration process naturally compensates for variations that would otherwise alter the trajectory of the outbreak, effectively stabilizing the projected outcomes.

However, this anchoring mechanism introduces specific compensatory dynamics when structural modifications are made to the model. Generally, introducing an epidemiological effect that delays disease progression, such as an incubation period, tends to spread the wave over a longer duration and flatten the peak. Because our model mathematically constrains the peak to remain at the scenario's midpoint, the calibration algorithm must counteract the delay induced by the latent phase by driving up the basic reproduction number (R_0).

Consequently, this requisite increase in R_0 to meet the temporal constraint leads to a much steeper and more pronounced epidemic wave. As illustrated in Figure E, incorporating an Exposed compartment in the SEIHR model to account for the incubation period results in a rapid escalation of projected daily hospital bed demand. The enhanced transmissibility required to achieve the mid-period peak ultimately overrides the natural flattening effect of the incubation phase, demonstrating how temporal anchoring shapes the model's structural response.

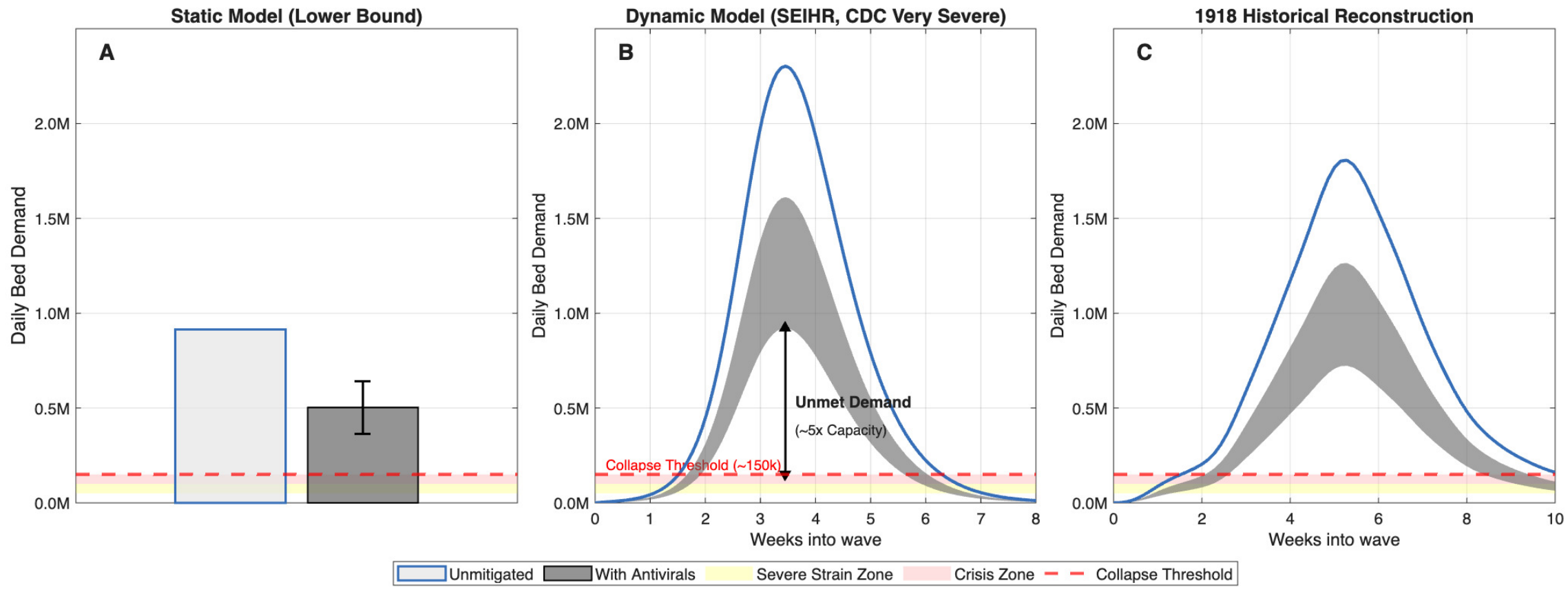

Figure E: Projected daily hospital bed demand under ”Very Severe” pandemic scenarios using an updated SHEIR model with an incubation period of 3 days. This updated simulation, contrasted against the SIHR model in Figure 1 of the main text, requires a higher calibrated basic reproduction number (R_0) to meet the fixed temporal constraint of a day 21 peak demand (the midpoint of a 6-week wave). This higher R_0 translates into a significantly more rapid and steeper escalation in daily bed demand, demonstrating how temporal anchoring amplifies the impact of structural model changes. The solid blue line represents unmitigated demand, which peaks much higher and earlier than in the SIHR model. Dark grey shaded regions show reduced demand with antivirals at 30–60% efficacy. Background colors and the red dashed line denote healthcare system stress levels and the collapse threshold based on national bed capacity (Table B and Figure 1 of the main text). Note that in this updated model, the surge is far more explosive, leading to healthcare system collapse even with optimal antiviral efficacy. Abbreviations: CDC, Centers for Disease Control and Prevention; SEIHR, Susceptible-Exposed-Infectious-Hospitalized-Recovered; SIHR, Susceptible-Infectious-Hospitalized-Recovered.

Reconstruction of Historical Hospital Demand

To provide an empirical benchmark for the planning scenarios, we reconstructed the daily hospital bed demand for the 1918 (H1N1), 1957 (H2N2), and 1968 (H3N2) influenza pandemics.

Data Selection and Standardization

We extracted weekly excess mortality estimates from [7, Figure 3], which were standardized to the 2015 US population ($N \approx 320$ million). As the 1918 pandemic propagated through distinct regional waves rather than as a simultaneous national event, we selected the US West Coast wave to serve as a representative morphological baseline. This regional wave exhibited an intermediate intensity profile, more acute than the Midwest but less peaked than the East Coast, providing a balanced template for a pandemic surge.

However, the source data, while derived from the West Coast, were normalized against the aggregate national population. To recover the specific wave intensity observed within the affected region, we applied a recalibration factor $\alpha = 0.24$, representing the West Coast's approximate share of the 2015 US population. Consequently, the source data were scaled by $1/\alpha$ to reconstruct the undiluted regional incidence rate.

Scenario Construction

We utilized the standardized West Coast mortality curve from [7] by assuming that the specific incidence rate and wave morphology observed in the West Coast region in 1918 would occur simultaneously across the entire United States population. This generates a high-magnitude surge comparable to the CDC "Very Severe" planning scenario.

For the 1957 and 1968 pandemics, the standardized mortality curves from [7] were used directly as national scenarios. The daily mortality rate $M(t)$ was obtained by dividing the weekly standardized counts by 7.

Transformation to Hospital Bed Demand

We estimated the continuous daily mortality curve using Piecewise Cubic Hermite Interpolating Polynomial (PCHIP) interpolation to preserve wave monotonicity. We then applied a deterministic back-calculation to convert mortality into hospital bed demand based on two conservative clinical assumptions: an in-hospital mortality rate (m_{hosp}) of 10% and a uniform length of stay (*LoS*) of 4 days.

Daily hospital admissions $A(t)$ were estimated as:

$$A(t) = \frac{M(t)}{m_{\text{hosp}}} = 10 \cdot M(t). \tag{5}$$

Daily hospital bed occupancy $H(t)$ was calculated as the convolution of admissions with the length-of-stay duration:

$$H(t) = \sum_{i=0}^{LoS-1} A(t-i). \tag{6}$$

References

[1] American Hospital Association. *AHA Hospital Statistics 2024 Edition*. AHA, Chicago, 2024.

[2] D. Blumenthal, E. J. Fowler, M. Abrams, and S. R. Collins. Covid-19 — implications for the health care system. *The New England Journal of Medicine*, 383:1483–1488, 2020.

[3] D. Carr et al. Resource burden of the 2009 influenza a (H1N1) pandemic in canadian hospitals. *Academic Emergency Medicine*, 2013.

[4] Department of Health. UK Influenza Pandemic Preparedness Strategy 2011, 2011. Available at GOV.UK.

[5] Department of Health (Northern Ireland). Northern ireland hospital statistics: Bed availability and occupancy. Technical report, Department of Health, Belfast, 2025.

[6] Seema Jain et al. Hospitalized patients with 2009 H1N1 influenza in the united states, april–june 2009. *The New England Journal of Medicine*, 2009.

[7] Martin I Meltzer, Manoj Gambhir, Charisma Y Atkins, and David L Swerdlow. Standardizing scenarios to assess the need to respond to an influenza pandemic. *Clinical Infectious Diseases*, 60(suppl_1):S1–S8, 2015.

[8] Ministry of Health, Labour and Welfare (MHLW). National Action Plan for Pandemic Influenza and New Infectious Diseases, 2013. Cabinet Decision, June 7, 2013.

[9] NHS England. Bed availability and occupancy data for q3 2025/26 (kh03). Technical report, NHS England, London, February 2026.

[10] Anne M. Presanis et al. Severity of the 2009 pandemic influenza a (H1N1) strain in the united kingdom. *PLOS ONE*, 2011.

[11] Public Health Scotland. Acute hospital activity and nhs beds information (2024/25). Technical report, Public Health Scotland, Edinburgh, 2025.

[12] U.S. Department of Health and Human Services (HHS) and Centers for Disease Control and Prevention (CDC). Pandemic Influenza Plan: 2017 Update, 2017.

[13] Welsh Government. Nhs beds: April 2023 to march 2024. Technical report, Welsh Government, Cardiff, August 2024.
